# Lack of parental rule-setting on eating is associated with a wide range of adolescent unhealthy eating behaviour both for boys and girls

**DOI:** 10.1186/s12889-016-3002-4

**Published:** 2016-04-27

**Authors:** Jana Holubcikova, Peter Kolarcik, Andrea Madarasova Geckova, Jitse P. van Dijk, Sijmen A. Reijneveld

**Affiliations:** Graduate School Kosice Institute for Society and Health, Faculty of Medicine, P. J. Safarik University, Trieda SNP 1, 040 11 Kosice, Slovakia; Department of Health Psychology, Faculty of Medicine, P. J. Safarik University, Trieda SNP 1, 040 11 Kosice, Slovakia; Olomouc University Society and Health Institute, Palacky University Olomouc, Křížkovského 8, 771 47 Olomouc, Czech Republic; Department of Community and Occupational Medicine, University Medical Center Groningen, University of Groningen, Hanzeplein 1, 9700 RB Groningen, Netherlands

## Abstract

**Background:**

Unhealthy eating habits in adolescence lead to a wide variety of health problems and disorders. The aim of this study was to assess the prevalence of absence of parental rules on eating and unhealthy eating behaviour and to explore the relationships between parental rules on eating and a wide range of unhealthy eating habits of boys and girls. We also explored the association of sociodemographic characteristics such as gender, family affluence or parental education with eating related parental rules and eating habits of adolescents.

**Methods:**

The data on 2765 adolescents aged 13–15 years (mean age: 14.4; 50.7 % boys) from the Slovak part of the Health Behaviour in School-Aged Children (HBSC) study 2014 were assessed. The associations between eating-related parental rules and unhealthy eating patterns using logistic regression were assessed using logistic regression.

**Results:**

Unhealthy eating habits occurred frequently among adolescents (range: 18.0 % reported skipping breakfast during weekends vs. 75.8 % for low vegetables intake). Of all adolescents, 20.5 % reported a lack of any parental rules on eating (breakfast not mandatory, meal in front of TV allowed, no rules about sweets and soft drinks). These adolescents were more likely to eat unhealthily, i.e. to skip breakfast on weekdays (odds ratio/95 % confidence interval: 5.33/4.15–6.84) and on weekends (2.66/2.12–3.34), to report low consumption of fruits (1.63/1.30–2.04) and vegetables (1.32/1.04–1.68), and the frequent consumption of sweets (1.59/1.30–1.94), soft drinks (1.93/1.56–2.38) and energy drinks (2.15/1.72–2.70).

**Conclusions:**

Parental rule-setting on eating is associated with eating behaviours of adolescents. Further research is needed to disentangle causality in this relationship. If causal, parents may be targeted to modify the eating habits of adolescents.

## Background

Unhealthy eating habits have been shown to be very common among adolescents in Europe [1–3]. Adolescents reporting unhealthy eating habits were at increased risk of becoming overweight [1] and suffering from fatigue, nutrient deficiencies, poor cognitive or physical performance [4], poorer mental health [5] and negative behavioural outcomes [6]. Adolescents who develop unhealthy eating habits are likely to continue this behaviour into adulthood [7], which can lead to a higher risk of many chronic illnesses. It is the reason why in recent years, particular attention has been devoted to nutritional behaviour during this period of life.

Parental rules have been shown to be one of the most important factors related to eating habits among adolescents [8–10]. The association between eating-related parental rules and eating habits of adolescents is particularly well-documented [10–15]. This suggests that parental rules on eating are a strong promoter of healthy eating patterns among adolescents, like more consumption of fruit and vegetables [11–14], regular consumption of breakfast [10, 15] and less frequent soft drinks intake [16]. The scope of the above-mentioned studies is limited in regard to specific unhealthy behaviours. There is a lack of studies investigating the wide range of behaviours of adolescents that contribute to the general profile of unhealthy eating among adolescents. Moreover, evidence on correlates of energy drink consumption, which is prevalent among adolescents, is scarce [17].

Several sociodemographic characteristics such as maternal education or parental modelling have previously been identified to have strong association with eating habits of adolescents. More specifically unhealthy eating habits of adolescents have been shown to be more likely in case of low maternal education, low socio-economic status and lack of parental rules on eating [18–20]. Eating behaviour of adolescents differed between rural and urban areas in some European countries which can be due to cultural and economic differences between rural and urban areas [21]. Finally, girls were in general found to have a healthier diet than boys. These gender differences in eating habits may be explained by girls’ greater focus on healthy eating [22].

The aim of our study was to assess the prevalence of parental rules on eating and the prevalence of a wide range of unhealthy eating behaviours among adolescents, to explore the associations between a lack of eating-related parental rules and various unhealthy eating habits, such as skipping breakfast, insufficient fruits and vegetables consumption, frequent sweets, soft drinks and energy drinks consumption of adolescents. Moreover we explored the association of sociodemographic characteristics such as gender, family affluence, completeness of family, parental education and urban context with eating related parental rules and eating habits of adolescents.

## Methods

### Sample and procedure

We used data from the Health Behaviour in School-aged Children (HBSC) study conducted in 2014 in Slovakia. To obtain a representative sample, we used a two-step sampling. In the first step, 151 larger and smaller elementary schools located in rural as well as in urban areas from all regions of Slovakia were asked to participate. These were randomly selected from a list of all eligible schools in Slovakia obtained from the Slovak Institute of Information and Prognosis for Education. This resulted in 130 schools agreeing to participate (86.1 %).

In the second step, we obtained data from 10,179 adolescents from the 5^th^ to 9^th^ grades of these schools (response rate: 78.8 %). Our final sample consisted of adolescents who responded to questionnaires that included measures on parental rules. In order to reduce the duration of administration of the questionnaires we used two types of questionnaire which partially consisted of the same questions. These overlapping questions regarded the mandatory questions that have to be included in any HBSC questionnaire. To these partially differing sets of other questions were added, which is allowed in the HBSC design. Not all adolescents administered the same version of questionnaire to avoid that the total questionnaire would become too long. Thus we created two different sets of questions (equal in length, but differing in variables included) - version A and version B with an equal share (50/50). Those version were randomly distributed within the sample. In this way, we reduced the sample of adolescents, leading to a final sample of 2765 adolescents (mean age: 14.38; 50.7 % boys).

The study was approved by the Ethics Committee of the Medical Faculty at the P.J. Safarik University in Kosice. Parents were informed about the study via the school administration and could opt out if they disagreed with their child’s participation. Participation in the study was fully voluntary and anonymous, with no explicit incentives provided for participation.

### Measures

Data for the present analyses were collected using questionnaires from the standardized research protocols for the 2014 WHO-collaborative HBSC study, with the aim of gaining insights into adolescents’ eating behaviour, eating-related parental rules and socio-demographic characteristics. Table 1 provides an overview of the measures used in this study.

**Table 1:** Description of items from the HBSC questionnaire used in this study

	Wording of the question	Options	Dichotomisation
Eating-related parental rules			
Breakfast mandatory	Breakfast is a mandatory part of the day in my family.	Always	<mostly
Meal in front of TV allowed	My parents allow me to eat (lunch and dinner) in front of the television or computer.	Mostly	
		Rarely	
Sweets and soft drinks consumption family rules	My parents allow me to eat sweets and drink sweetened beverages (e.g. Coca Cola, Fanta…) when I want and how much I want.	Never	
Eating patterns			
Breakfast on weekends	How often do you usually have breakfast (more than a glass of milk or fruit juice)?	I never have breakfast	<five weekdays
Breakfast on weekdays	Please tick one box for weekdays and one box for weekend	One day	<two days (weekend)
		Two days	
		Three days	
		Four days	
		Five days	
Low fruit consumption	How many times a week do you usually eat or drink….?	Never	Fruits and vegetables consumption
Low vegetables consumption		Less than once a week	<once a day
High soft drinks consumption		Once a week	Soft drinks and sweets consumption
High sweets consumption		2–4 days a week	>5–6 days a week
High energy drinks consumption		5–6 days a week	
		Once a day, every day	Energy drinks consumption > once a week
		Every day, more than once	
Perceived family wealth			
Low family affluence	How well off do you think your family is?	Very well off	<average
		Quite well off	
		Average	
		Not so well off	
		Not at all well off	

### Statistical analyses

Statistical analyses were performed using IBM SPSS statistics 20.0 for Windows. Firstly, the prevalence of parental rules and eating habits of adolescents were calculated for total sample and stratified by gender and family affluence. Differences between boys and girls and adolescents reporting low family affluence and others were tested using Chi-square tests. Secondly, the associations of sociodemographic characteristics (gender, family affluence, completeness of family, parental education and urban context) with parental rules and eating habits of adolescents were assessed. Thirdly, we assessed the associations between eating-related parental rules and unhealthy eating patterns using logistic regression. We adjusted these analyses for gender and family affluence.

## Results

Over 20 % of the sample reported no parental rules on eating. Actually more than a half of adolescents admitted the absence of one of the following rules in their families: mandatory breakfast, meal in front of TV not allowed or no rules about sweets and soft drinks consumption. The prevalence of unhealthy eating behaviours among adolescents varied from 18 % for skipping breakfast during the weekends to 75 % for low vegetables consumption (Table 2).

**Table 2:** Description of the sample - parental rules on eating and unhealthy eating habits for the total sample and stratified by gender and family affluence

	Total	Boys	Girls	*P* value	Low family affluence	Higher family affluence	*P* value
	*N* = 2765 (%)	*N* = 1402 (%)	*N* = 1363 (%)		*N* = 134 (%)	*N* = 2571 (%)	
Parental rules							
No eating-related parental rules	524 (20.5)	212 (16.7)	312 (24.2)	0.000	38 (29.9)	484 (20.1)	0.008
Breakfast not mandatory	1324 (51.1)	601 (46.7)	723 (55.5)	0.000	81 (62.8)	1230 (50.5)	0.006
Meal in front of TV allowed	1420 (54.6)	698 (54.2)	722 (55.0)	0.652	77 (60.2)	1335 (54.5)	0.213
No rules about sweets and soft drinks	1421 (54.3)	723 (55.5)	698 (53.2)	0.232	79 (60.8)	1336 (54.3)	0.148
Eating behaviours							
Skip breakfast weekend	489 (18.0)	240 (17.5)	249 (18.4)	0.541	36 (27.5)	440 (17.4)	0.003
Skip breakfast weekday	1511 (55.4)	711 (51.6)	800 (59.4)	0.000	89 (66.9)	1393 (54.9)	0.006
Low fruit consumption	1894 (69.6)	1022 (74.5)	872 (64.6)	0.000	98 (74.2)	1759 (69.4)	0.237
Low vegetables consumption	2042 (75.8)	1100 (80.6)	942 (70.8)	0.000	102 (77.9)	1892 (75.4)	0.529
High sweets consumption	973 (36.2)	445 (32.9)	528 (39.6)	0.000	50 (38.8)	905 (36.1)	0.546
High soft drinks consumption	697 (25.6)	381 (27.6)	316 (23.5)	0.014	32 (24.4)	649 (25.6)	0.760
High energy drinks consumption	604 (22.2)	395 (28.8)	209 (15.5)	0.000	25 (18.9)	560 (22.1)	0.395

Missing values N(%): No eating-related parental rules 207 (7.5 %), Breakfast not mandatory 175 (6.3 %), Meal in front of TV allowed 164 (5.9 %), No rules about sweets and soft drinks 149 (5.4 %), Skip breakfast weekend 47 (1.7 %), Skip breakfast weekday 39 (1.4 %), Low fruit consumption 45 (1.6 %), Low vegetables consumption 71 (2.6 %), Sweets every day 79 (2.9 %), Soft drinks every day 45 (1.6 %), Energy drinks regularly 44 (1.6 %). Low family affluence 60 (2.2 %)

Boys were less likely to report the lack of eating related parental rules and to skip breakfast during weekdays than girls, but their chance to low fruit and vegetables consumption, high soft drinks and energy drinks consumption was higher than girls. Adolescents reporting low family affluence reported significantly more often the lack of parental rules and skipping breakfast but they did not differed in the prevalence of other unhealthy eating habits from their peers with medium of high family affluence. Adolescents from incomplete families were more likely to report a lack of parental rules and skipping breakfast during weekdays. Adolescents living in rural areas were less likely to report a lack of parental rules and more likely to report a low fruit and high sweets consumption, and a high soft drinks and energy drinks consumption compared to adolescents from urban areas. Low education of mother and father was positively associated with a lack of parental rules, skipping breakfast, low fruit consumption and high sweets, soft drinks, and energy drinks consumption (Table 3).

**Table 3:** The association of gender, family affluence, family completeness, urban context and parents’ educational level with eating-related parental rules and unhealthy eating habits among adolescents; odds ratios (OR), and 95 % confidence intervals (CI) between parentheses

	No eating-related parental rules OR (95 % CI)	Skip breakfast weekend OR (95 % CI)	Skip breakfast weekday OR (95 % CI)	Low fruit consumption OR (95 % CI)	Low vegetables consumption OR (95 % CI)	High sweets consumption OR (95 % CI)	High soft drinks consumption OR (95 % CI)	High energy drinks consumption OR (95 % CI)
Gender - boy	0.62 (0.51–0.76)***	ns	0.72 (0.62–0.84)***	1.60 (1,35–1.88)***	1.71 (1.43–2.05)***	ns	1.24 (1.04–1.47)*	2.20 (1.82–2.65)***
Family affluence – low	1.69 (1.14–2.51)**	1.80 (1.21–2.67)**	1.66 (1.14–2.40)**	ns	ns	ns	ns	ns
Incomplete family	1.43 (1.03–1.98)*	ns	1.39 (1.05–1.84)*	ns	ns	ns	ns	ns
Rural area	0.75 (0.61–0.92)**	ns	ns	1.30 (1.09–1.54)**	ns	1.18 (1.01–1.39)*	1.34 (1.12–1.60)**	1.22 (1.01–1.46)*
Mother education – low	1.30 (1.04–1.61)*	1.64 (1.31–2.05)***	1.25 (1.05–1.49)*	1.42 (1.17–1.73)***	ns	1.37 (1.14–1.64)**	1.67 (1.37–2.03)***	1.80 (1.46–2.21)***
Father education – low	1.33 (1.07–1.64)**	1.50 (1.27–1.78)***	1.50 (1.27–1.78)***	1.37 (1.14–1.65)**	ns	1.34 (1.12–1.60)**	1.82 (1.50–2.21)***	1.81 (1.48–2.23)***

*p < 0.05, **p < 0.01, ***p < 0.001, ns = not statistically significant at level *p* < 0.05

The results of the multiple logistic regressions showed that adolescents who reported a lack of eating-related parental rules were more likely to eat unhealthily . In the analyses of each eating-related rule separately, we found that unhealthy eating habits among adolescents were associated with an absence of these parental rules. Only low consumption of fruit and vegetables showed no relationship with some rules on eating. Adding gender and perceived family wealth into the models did not affect the strength of the association of rule setting on eating and the unhealthy eating habits of adolescents (Table 4). When exploring clustering by class and school, multilevel analyses showed a very small and non-significant clustering and yielded identical or nearly identical ORs and 95 % CIs.

**Table 4:** The association between eating-related parental rules and unhealthy eating habits among adolescents adjusted for gender and perceived family wealth; odds ratios (OR), and 95 % confidence intervals (CI) between parentheses

	Skip breakfast weekend OR (95 % CI)	Skip breakfast weekday OR (95 % CI)	Low fruit consumption OR (95 % CI)	Low vegetables consumption OR (95 % CI)	High sweets consumption OR (95 % CI)	High soft drinks consumption OR (95 % CI)	High energy drinks consumption OR (95 % CI)
No eating-related parental rules	2.66 (2.12–3.34)*	5.33 (4.15–6.84)*	1.63 (1.30–2.04)*	1.32 (1.04–1.68)*	1.59 (1.30–1.94)*	1.93 (1.56–2.38)*	2.15 (1.72–2.70)*
Breakfast not mandatory	2.30 (1.85–2.86)*	13.21 (10.90–16.02)*	1.37 (1.15–1.63)*	1.34 (1.12–1.62)*	1.20 (1.02–1.41)*	1.42 (1.18–1.70)*	1.66 (1.36–2.02)*
Meal in front of TV allowed	1.64 (1.33–2.03)*	1.18 (1.01–1.38)*	ns	ns	1.30 (1.10–1.53)*	1.56 (1.29–1.87)*	1.74 (1.43–2.12)*
No rules about sweets and soft drinks	1.58 (1.28–1.95)*	1.27 (1.08–1.49)*	1.40 (1.18–1.66)*	ns	1.54 (1.30–1.82)*	2.67 (2.20–3.23)*	2.21 (1.80–2.71)*

*p < 0.05, ns = not statistically significant at level *p* < 0.05

## Discussion

We found that more than 20 % of adolescents reported having no parental rules on eating, and the prevalence of unhealthy eating habits varied between 18 % for skipping breakfast during weekends to 75 % for low consumption of vegetables. A lack of parental rule-setting on eating was strongly associated with unhealthy eating habits among boys and girls. Several sociodemographic characteristics such as gender, family affluence, family structure, urban context and education of parents were related to unhealthy eating habits of adolescents and to lack of parental rules on eating.

More than 20 % of the adolescents perceived no parental rules on eating. Previous evidence focused rather on the association of the presence of parental rules and unhealthy eating habits; literature dealing with the absence of parental rules on eating is scarce. Our further findings suggest that unhealthy eating habits, such as skipping breakfast, low fruit and vegetable consumption, frequent sweets, soft drinks and energy drinks consumption, were very frequent among adolescents. Compared with the findings of the previous HBSC survey in 2009, the prevalence of fruit and vegetable consumption and breakfast skipping did not change importantly, and the prevalence of sweets and soft drinks consumption decreased slightly [3]. The consumption of energy drinks among adolescents was found to be considerably higher than existing findings from Europe suggest [23]. The high prevalence of unhealthy eating habits among adolescents may reflect the limited effectiveness of preventive strategies aimed at improving their eating habits. Generally, these strategies take place in the school environment, which may be the reason of their limited effectiveness. In line with this, Lindsay et al. [8] highlight the success of interventions within a variety of settings, including schools, health services and the family setting, and emphasize the critical role of parents in these interventions.

Adolescents who perceived a lack of parental rules on eating were at higher risk to eat unhealthily. Those who reported an absence of one of three examined parental rules were also at risk of unhealthy eating, but the chances were lower. Our findings confirm previous evidence about the connection between parental rule-setting on eating and unhealthy behaviour of adolescents [11–14, 16]. In addition, the present findings suggest that when the number of parental rules was reduced, the prevalence of unhealthy eating behaviours increased. Taking into account that parents have been shown to have a crucial role in shaping their children’s dietary practices [8, 9], our findings indicate this to be a possible factor contributing to unhealthy eating habits among adolescents. Given the cross-sectional design of present study, the findings do not imply a causal path. An alternative explanation could be that having parental rules are an expression of more general family food and eating practices which may determine both parental rules and eating habits of adolescents. This is definitely of interest for further research.

Gender was found to have an important association with eating habits of adolescents. Although boys reported a lack of parental rules on eating less often, they were more likely to eat unhealthily than girls. This can point at gender differences regarding the degree of obedience of adolescents to these rules – boys may perceive the existence of the rule but may not follow it. On the other side, given the vulnerability of boys to eat unhealthy [3], parental rules on eating may be stricter in boys than in girls.

Our findings indicate that several sociodemographic characteristics such as family affluence, family structure, urban context and educational level of parents were associated with parental rule setting on eating and also with eating habits of adolescents. Adolescents reporting low family affluence, incomplete family, low parental education, living in a rural area and a lower socioeconomic status were at higher risk of unhealthy eating habits which is in line with previous findings [3, 24]. We explored this relationship using several indicators related to socioeconomic status (such as education of parents) to ascertain the validity of the results.

### Strengths and limitations

The major strengths of our study are its large sample and representativeness for Slovak adolescents and its high response rate. In addition, the measures of food consumption frequency used in the present study have been well-validated [25] and extensively used in HBSC surveys. We studied a wide range of unhealthy eating habits in the context of parental rules on eating. However, some limitations should be also noted. Firstly, we used a cross-sectional design; thus, no final causal conclusion can be drawn. Second, our data were based on self-reports of adolescents, which are based on the subjective perception of the adolescent including his or her disobedience to rules and may also be influenced by social desirability. Moreover, the measures of parental rules were based on subjective perception which can reflect rather the obedience degree of the adolescents to these rules. However considerable proportion of adolescents do not follow the rules which their parents apply based on their reports, so obedience bias this measurement only marginal. Thirdly, analyses provided in this study targeted the main relationship between parental rule-setting on eating and unhealthy eating of adolescents; we did not address the role of biological and psycho-social factors which can influence this relationship.

### Implications

The frequent lack of parental rule-setting on eating and the high prevalence of unhealthy eating habits among adolescents indicate the need for interventions carried out in family settings. The findings of the present study on the connection between a lack of parental rule-setting on eating and unhealthy eating habits of adolescents suggest that reinforcing parental rule-setting on eating may improve adolescent eating habits especially among boys and among adolescents with low socioeconomic status.

The associations of a lack of parental rule-setting on eating with unhealthy eating habits of adolescents requires further study to disentangle causality and the mechanisms behind the connection between parental rule-setting and adolescent behaviour. Longitudinal and experimental study designs are needed for this. Moreover objective measures of eating habits (such as anthropometric measures, body composition or body fat) should be explored in future research of family context of unhealthy eating habits of adolescents.

## Conclusions

This study demonstrated an association between a frequent lack of parental rules on eating and the high prevalence of a wide range of unhealthy eating habits among boys and girls. Adolescents perceiving the lack of parental rules on eating were at higher risk of unhealthy eating habits. Public health strategies should address family eating practices.

## Availability of data and materials

The dataset supporting the conclusions of this article is available upon request.

## Abbreviations

CIs: confidence intervals
HBSC: Health Behaviour in School-aged Children
ORs: odds ratios
SPSS: Statistical Package for the Social Sciences
